# Benzo[k,l]xanthene Lignan-Loaded Solid Lipid Nanoparticles for Topical Application: A Preliminary Study

**DOI:** 10.3390/molecules27185887

**Published:** 2022-09-10

**Authors:** Cristina Torrisi, Nunzio Cardullo, Stefano Russo, Alfonsina La Mantia, Rosaria Acquaviva, Vera Muccilli, Francesco Castelli, Maria Grazia Sarpietro

**Affiliations:** 1 Department of Drug and Health Sciences, University of Catania, Viale Andrea Doria 6, 95125 Catania, Italy; 2 Department of Chemical Sciences, University of Catania, Viale Andrea Doria 6, 95125 Catania, Italy

**Keywords:** SLN, benzo[k,l]xanthene lignans, DSC, biomembrane model, HFF-1

## Abstract

Skin is the first human barrier that is daily exposed to a broad spectrum of physical and chemical agents, which can increase reactive oxygen species (ROS) and lead to the formation of topical disorders. Antioxidant molecules, such as benzo[k,l]xanthene lignans (BXL), are ideal candidates to eliminate or minimize the effects of ROS. Herein, we aimed to formulate BXL-loaded solid lipid nanoparticles (SLN-BXL) to improve the bioavailability and interaction with the skin, and also to investigate the protective impact against intracellular ROS generation in HFF-1 in comparison with the drug-free situation. SLN-BXL were formulated using the PIT/ultrasonication method, and then were subjected to physicochemical characterizations, i.e., average size, zeta potential (ZP), polydispersity index (PDI), encapsulation efficiency (%EE), thermotropic behavior, and interaction with a biomembrane model. The results show a mean size around 200 nm, PDI of 0.2, and zeta potential of about −28 mV, with values almost unchanged over a period of three months, while the EE% is ≈70%. Moreover, SLN-BXL are able to deeply interact with the biomembrane model, and to achieve a double-action release in mildly hydrophobic matrices; the results of the in vitro experiments confirm that SLN-BXL are cell-safe and capable of attenuating the IL-2-induced high ROS levels. In conclusion, based on our findings, the formulation can be proposed as a candidate for a preventive remedy against skin disorders induced by increased levels of ROS.

## 1. Introduction

Skin is the largest organ and covers the entire surface of the human body. Anatomically, it is composed of different layers—epidermis, dermis, and hypodermis—of which the outermost is the stratum corneum (SC). The latter represents the first protective human physical barrier: it prevents the loss of water and electrolytes, reduces the penetration of chemical substances, and protects against pathogenic microorganisms [1]. Skin has, historically, been used for the topical delivery of compounds since it reduces the risk of systemic side effects and allows the drug to remain concentrated in the targeted tissue. However, skin is composed of alternated hydrophobic and hydrophilic layers (ranging from the perspiratio insensibilis to the adipocytes), and the particular structure of SC is comparable to a brick wall made of these sheets, so it is not surprising that only a few substances, with an ideal n-octanol/water partition ratio (logP), are able to cross the skin [2]. A way to overcome this problem, and improve drug penetration and distribution, is the employment of lipid nanoparticles as carriers of active molecules [3]. Solid lipid nanoparticles (SLN) have been studied as alternative colloidal carriers to the traditional methods, especially for the delivery of lipophilic compounds. They are composed of a solid lipid matrix dispersed in aqueous medium and stabilized by various surfactants. Cutaneous use of SLN exhibits several advantages, such as biocompatibility (since they are composed of physiological and biodegradable lipids), protection of encapsulated compounds sensitive to light, oxidation and hydrolysis, and drug release control [4]. The strategic mechanism behind lipid nanoparticles for topical application is the formation of an occlusive film at the SC surface that prevents water loss and improves the skin lipid barrier, leading to corneocytes packing reduction and inter-corneocyte gaps enlargement, with a consequent increase in drug penetration [5,6,7]. It was also observed that a better SC occlusion and a greater interaction with skin is obtained with smaller lipid nanoparticles, generally around 200–300 nm [8]. Nowadays, skin is constantly exposed to environmental stresses (ultraviolet radiations, chemical substances, and pathogens) which leads to the formation of ROS species. The latter can activate proliferative and cell survival signals that can alter apoptotic pathways and lead to an extensive number of skin disorders. One way to eliminate or minimize the effects of ROS and/or their products, reducing oxidative damage to cellular constituents, is the use of antioxidant molecules [9].

**Scheme 1:**
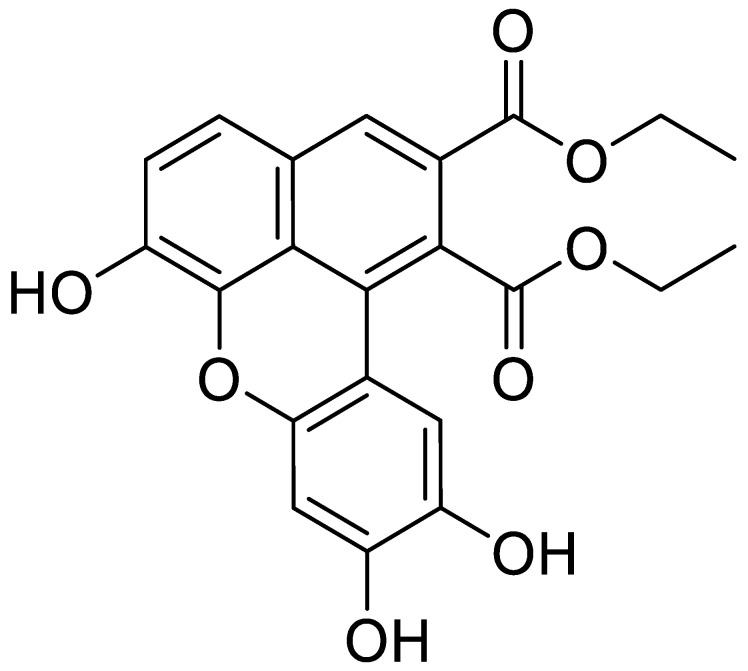
BXL structure.

In the present work, a bioinspired lignan (BXL, Scheme 1), belonging to the rare group of benzoxanthene lignans, was used to prepare SLN formulations that will be used in the preventive treatment of the skin conditions.

BXL and other analogues show an array of biological properties, including antioxidant, antifungal [10], antibacterial [11], anti-inflammatory [12], anti-proliferative, anti-angiogenic, DNA-binding [13], and proteasome-inhibiting activities [14,15]. However, these bioactive compounds are very lipophilic (BXL: capacity factor 3.24; calculated logP 3.71) [16] and their potential use in pharmacological studies is limited, due to the very low solubility in aqueous media (See Figure S1, Supplementary Material for the predicted physicochemical properties of BXL) [17,18]. The introduction of BXL into SLN produced promising results in terms of size, polydispersity index, zeta potential, stability, and entrapment efficiency, while retaining its antioxidant and anti-inflammatory activity [19].

The interest behind the possible applications of this uncommon family of molecules drove us into further research. In this study, we employ a new SLN formulation with and without BXL, aiming for better encapsulation and less cytotoxicity (compared to the previous formulation), which we tested and validated from the calorimetric, technological, and biochemical perspectives.

## 2. Results and Discussion

### 2.1. SLN Characterization

**Figure 1:**
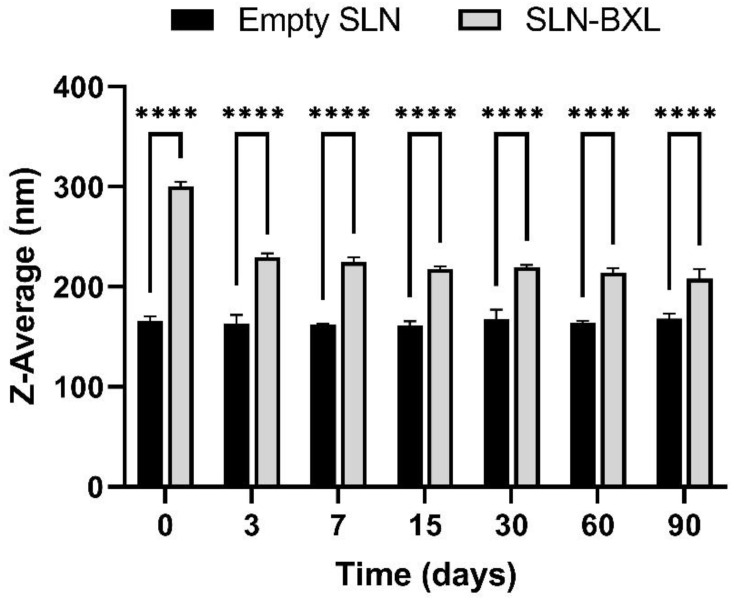
SLN mean particle size. The results are shown as mean ± standard deviation. Confidence intervals calculated by two-way ANOVA: **** = *p* < 0.0001.

**Figure 2:**
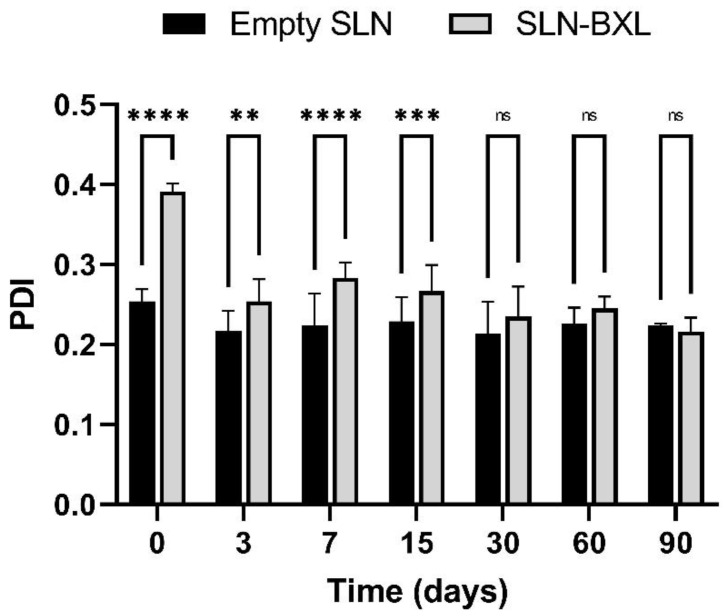
SLN polydispersity index (PDI). The results are shown as mean ± standard deviation. Confidence intervals calculated by two-way ANOVA: ns = not significant; ** = *p* < 0.01; *** = *p* < 0.001; **** = *p* < 0.0001.

**Figure 3:**
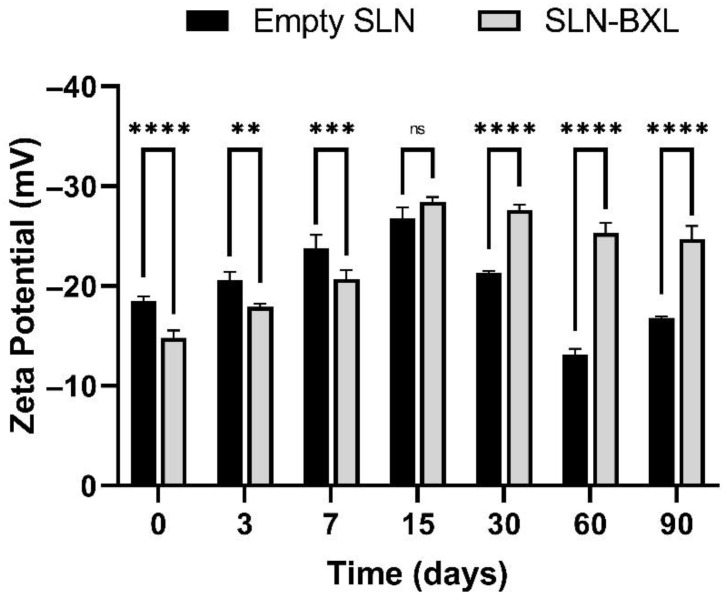
SLN zeta potential. The results are shown as mean ± standard deviation. Confidence intervals calculated by two-way ANOVA: ns = not significant; ** = *p* < 0.01; *** = *p* < 0.001; **** = *p* < 0.0001.

In order to characterize and evaluate the physiochemical stability of the produced solid lipid nanoparticles, mean particle size (Figure 1), polydispersity index (PDI) (Figure 2), and zeta potential (Figure 3) were analyzed over three months at 25 °C.

Empty SLN exhibit a mean particle size of 165 nm, remain unchanged over the period, while the association with the BXL causes an initial size increase at 300 nm, probably caused by the adsorption [20] of BXL onto the SLN surface, and/or a slow, gradual distribution of the compound into the nanoparticles matrix; after 3 days, the SLN-BXL z-average stabilizes at 220 nm. The mean particle size values are additionally confirmed by PDI values, which are initially high and then stabilize around 0.2.

Regarding zeta potential, during the first 15 days, both unloaded and loaded SLN show an increment of said absolute value up to −28 mV. Following that day, unloaded SLN undergoes a non-linear decrement of ZP whereas SLN-BXL remains almost unchanged, possibly indicating a balancing effect of BXL on the electrical behavior of SLN.

The results obtained suggest that the preparation is suitable for BXL encapsulation and delivery from a pharmaceutical technology standpoint, since mean size and PDI are nearly unaffected by the compound inclusion, retaining their ideal small values, while zeta potential is even positively influenced and brought to higher absolute values, causing a net negative charge-driven separation between nanoparticles and, thus, avoiding caking phenomena [21]—all in a time period as long as 3 months without usage of preservatives.

### 2.2. Encapsulation Efficiency

The previously developed methodology, described in [19], based on Sephadex LH20 column chromatography followed by high pressure liquid chromatography—UV quantitative analysis, allows the determination of the entrapment efficiency (EE%) of the SLN. The details are reported in the experimental section. The EE% can be calculated indirectly (see Equations) by quantification of free BXL eluted with acetone from Sephadex LH20, and directly by recovering the SLN-BXL from aqueous eluate. The results achieved with the two equations agree with each other, as the EE% values are between 69.4 and 74.2%. Of note, the formulation employed in this study leads to an increase in encapsulation efficiency (calculated with Equation (1)) compared to that observed in the previous work.

These higher, but not total, encapsulation values provide more control in BXL release over time when in contact with lipophilic matrices such as cellular membranes, diminishing, although not denying, a burst release imputable to free BXL. For a possible therapeutical application, optimal employment of this suspension should be as a whole, so that a rapid onset effect, along with slower long-lasting release, is obtained [22].

### 2.3. Drug Release

**Figure 4:**
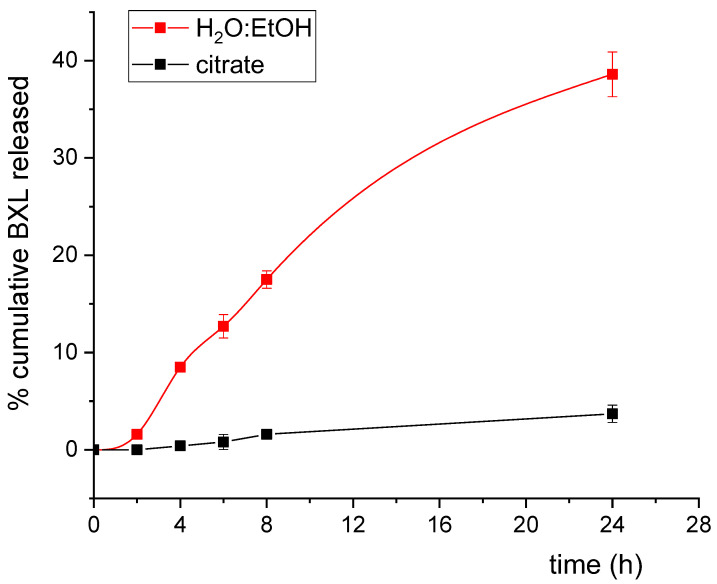
In vitro BXL release profile in citrate buffer (pH 5; black line) and in H_2_O:EtOH 80:20 mixture (red line). The data plotted are means (*n* = 3) ± SD.

The in vitro release of BXL from SLN is achieved by dialysis method in citrate buffer (pH 5), TRIS buffer (pH 7.4), and H_2_O/EtOH (80:20). The BXL released within 24 h was assayed by HPLC–UV. The results are reported in Figure 4 as % of cumulative BXL released vs. time. As shown by the black line, the release in citrate buffer is slow, reaching only 3.7% at 24 h, and the release in TRIS buffer shows nearly the same trend. When BXL-SLN are dispersed in H_2_O/EtOH, the release is faster within 24 h, reaching up to 38.6%.

Since the whole formulation was used, these data suggest that in strongly hydrophilic environments, such as blood (pH 7.4) and acid urine (pH 5), both encapsulated and free BXL remain in and adsorbed onto nanoparticles, respectively (pointing to a possible higher half-life and slower excretion), whilst a broader, faster release over time is observed when SLN come in contact with slightly more lipophilic substrates, such as the proposed H_2_O/EtOH mixture model (needed for sufficient solubility conditions, since both SLN and BXL are not soluble solely in water) [23] or, as we will see successively, the water-suspended biomembrane model based on DMPC MLV.

### 2.4. Cell Viability

**Figure 5:**
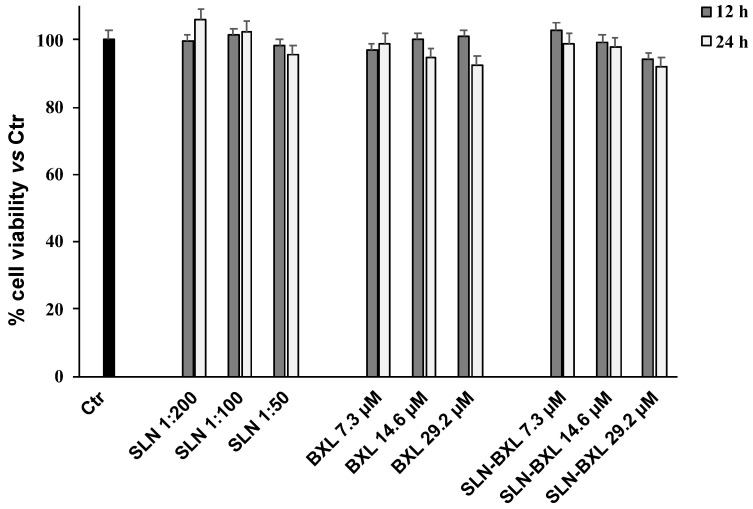
Cell viability in HFF-1 untreated (Ctr) and treated with empty SLN, BXL, and SLN-BXL at different concentrations. Values are expressed as mean ± S.D. of four experiments in triplicate.

SLN-BXL treatment does not affect the viability of HFF-1 cells in any concentrations tested after 12 h and 24 h of exposure. BXL and empty SLN also show no toxicity in HFF-1 cells in the same experimental conditions (Figure 5). Since the administration of SLN-BXL, BXL, and empty SLN at 12 h and 24 h induce a similar effect, we have chosen to use 12 h as exposure time for ROS assay.

### 2.5. ROS Determination

**Figure 6:**
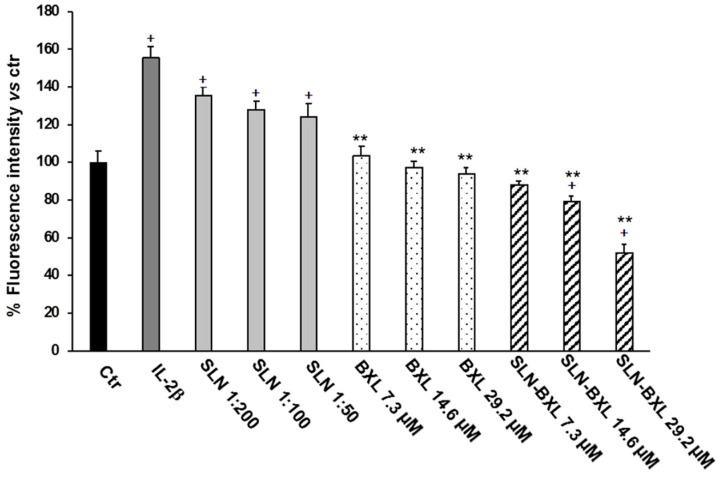
ROS levels in HFF-1 cells untreated (Ctr), treated with IL-2β (3 µM), and pre-treated with SLN, BXL, and SLN-BXL at different concentrations. Values are expressed as mean ± S.D. of four experiments in triplicate. + Significant vs. untreated control cells *p* < 0.001; ** significant vs. IL-2β-treated cells: *p* < 0.001.

Figure 6 shows that exposure of HFF-1 cells to IL-2β (3 μM) for 12 h increases ROS levels by approximately 50% relative to the control, as revealed by the intensity of the fluorescence; pre-treatment with SLN-BXL can induce, in a dose-dependent manner, a decrease in radical species compared to the cells treated with IL-2β and to the control. In particular, SLN-BXL is more effective than BXL alone, and, at a concentration of 29.2 μM, reduces ROS levels by about 50% compared to untreated cells.

These results may be due to a possible modification of the surface of SLN that allows a greater release of BXL. Furthermore, the data reported in Figure 6 indicate that empty SLN, while not altering cell viability, slightly increase ROS levels compared to control, unlike SLN-BXL. The SLN-BXL formulation, in fact, is able to block the ROS levels induced by IL-2β, and the encapsulation with SLN enhances the antioxidant effect of BXL.

Recently, it was reported that oxidative stress can induce skin damage due to a decrease in the levels of endogenous antioxidants [24,25,26]. Increased levels of ROS can, indeed, be responsible for an altered cellular redox state, DNA damage, and release of pro-inflammatory processes.

The results of the in vitro experiments confirm that the increased ROS levels due to IL-2β treatment in HFF-1 cells are neutralized by SLN-BXL, which sport a stronger antioxidant activity than BXL alone; SLN can then be used to improve the overall efficacy of the compound.

### 2.6. Empty SLN and SLN-BXL Calorimetric Analysis

**Figure 7:**
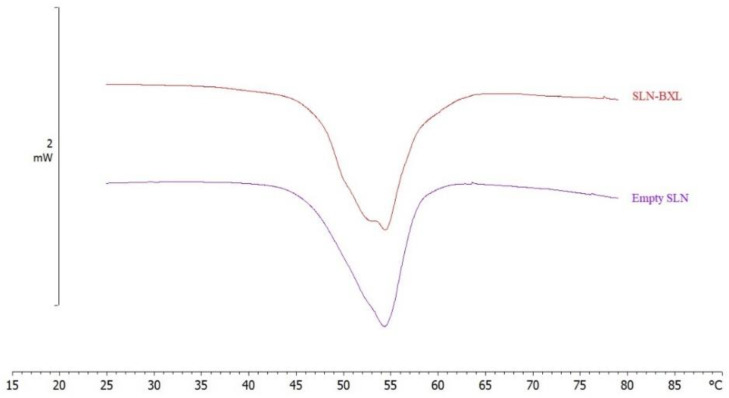
Calorimetric curves, in heating mode, of SLN and SLN-BXL.

The thermotropic behavior of the SLN was evaluated by DSC analysis (Figure 7). The calorimetric curve of empty SLN is characterized by a main peak at 54.40 °C and a shoulder on lower temperature. In the SLN-BXL calorimetric curve, as the temperature increases, a smaller shoulder, a secondary peak at 52.80 °C, and the primary peak at 54.60 °C are observed. The prominent differences among the empty and SLN-BXL calorimetric curves suggest that BXL is inside the SLN structure, affecting its response to heat and temperature-based transitions.

### 2.7. MLV/SLN Interaction Study

Differential scanning calorimetry was used to evaluate an eventual interaction between a biomembrane model made of DMPC MLV and empty SLN or SLN-BXL.

**Figure 8:**
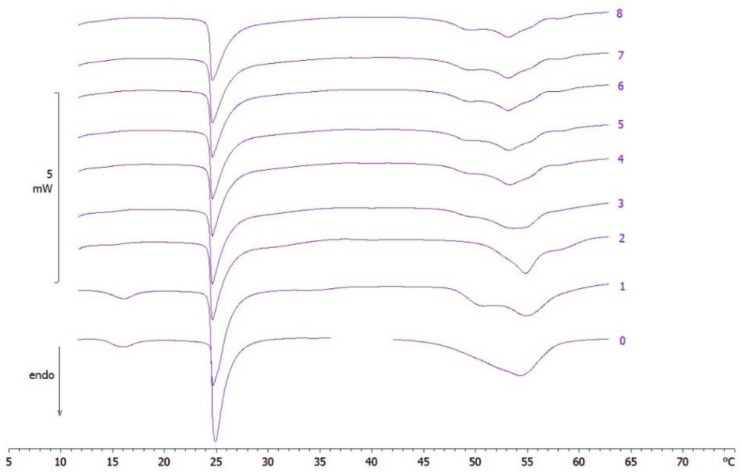
Calorimetric curves, in heating mode, of MLV put in contact with empty SLN, at pH 5, at increasing incubation time. The numbers on the right side of the curves refer to the calorimetric scans. The curves 0 refer to the samples before contact.

**Figure 9:**
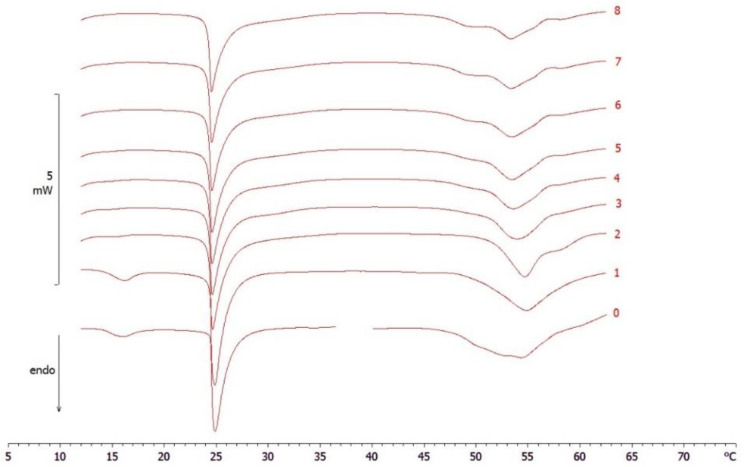
Calorimetric curves, in heating mode, of MLV put in contact with SLN-BXL at pH 5, at increasing incubation time. The numbers on the right side of the curves refer to the calorimetric scans. The curves 0 refer to the samples before contact.

**Figure 10:**
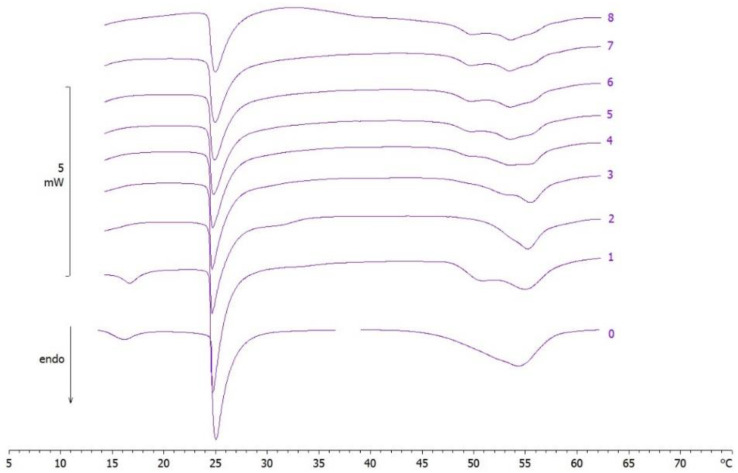
Calorimetric curves, in heating mode, of MLV put in contact with empty SLN, at pH 7.4, at increasing incubation time. The numbers on the right side of the curves refer to the calorimetric scans. The curves 0 refer to the samples before contact.

**Figure 11:**
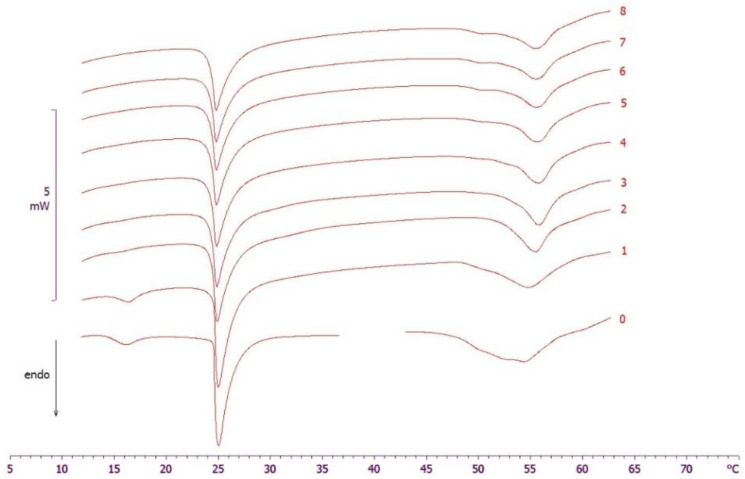
Calorimetric curves, in heating mode, of MLV put in contact with SLN-BXL, at pH 7.4, at increasing incubation time. The numbers on the right side of the curves refer to the calorimetric scans. The curves 0 refer to the samples before contact.

The interaction was assessed both at pH 5.0 (Figure 8 and Figure 9) and pH 7.4 (Figure 10 and Figure 11). The interaction between MLV and SLN is evidenced by the variation of the calorimetric peaks of MLV and/or SLN. The calorimetric curve of MLV shows a pre-transition peak at about 17 °C, related to the transition from the ordered gel phase to the ripple phase, and a main peak at about 25 °C, related to the transition from the ripple phase to the disordered liquid crystalline phase [27]. The contact of SLN with MLV induces several variations in the calorimetric peaks of MLV and SLN, in both pH conditions.

Regarding the experiment carried out with empty SLN and MLV, the pre-transition peak of MLV disappears and the main peak becomes smaller as the contact time increases; the calorimetric peaks of SLN vary significatively in terms of enthalpy and morphology. In the experiments carried out with SLN-BXL, the calorimetric curve of MLV loses the pre-transition peak, whereas the main peak becomes smaller; the calorimetric curve of SLN-BXL also undergoes evident variations. 

Experimental results indicate a strong, pH-independent interaction between both SLN preparations and the MLV biomembrane model that can be supposed to be of penetrative nature, since changes in enthalpy and morphology are far more prominent than temperature-based ones [28]. Moreover, it can be observed, especially at pH 5, that on late scans the calorimetric morphology of both SLN specimens tested becomes gradually similar to practically superimposable, hinting at a complete redistribution of BXL in the MLV matrix and, thus, to a behavioral alignment of SLN-BXL back to empty SLN after the interaction.

## 3. Materials and Methods

### 3.1. Materials

Precirol^®^ ATO 5 (Glyceryl Distearate) was kindly donated by Gattefossé (Saint-Pries, France). Tween^®^ 80 (Polysorbate 80) was purchased from Sigma Aldrich Co. (St. Louis, MO, USA). Dimyristoylphosphatidylcholine (DMPC) was obtained from Genzyme (Liestal, Switzerland).

Caffeic acid, Mn(OAc)3* 2 H_2_O, 3-(4,5-dimethyl-2-thiazolyl)-2,5-diphenyl-2H-tetrazolium bromide (MTT), Dulbecco’s modified Eagle’s medium, fetal bovine serum, glucose, penicillin–streptomycin, interleukin-2 (IL-2), and 2′,7′-dichlorofluorescein diacetate were purchased from Sigma Aldrich (Milan, Italy).

Human foreskin fibroblast (HFF-1) cells were obtained from ATCC^®^ SCRC-1041 TM (ATCC, Manassas, VA, USA). Purified water from Millipore-Q^®^ Gradient A10TM ultra-pure water system (Millipore, Guyancourt, France) was used throughout the study.

In the manuscript, the benzoxanthene lignan (diethyl-6,9,10-trihydroxybenzo[k,l]xanthene-1,2-dicarboxylate, indicated as BXL) employed in the formulations was obtained as described previously, and ^1^H NMR and HPLC–UV analyses established its purity [16].

### 3.2. SLN Preparation

SLN were prepared by combined PIT and ultrasonication methods, following the procedures reported elsewhere [29,30,31]. Briefly, lipid phase (consisting of 1.5% *w*/*w* Precirol^®^ ATO 5 for empty SLN; 1.5% *w*/*w* Precirol^®^ ATO 5 plus 0.06% *w*/*w* BXL for SLN-BXL) and aqueous phase (consisting of 0.5% *w*/*w* Tween^®^ 80 in water) was separately heated at 75 °C on a magnetic heat plate, then the aqueous phase was added drop by drop, at constant temperature and under stirring (300–400 rpm), to the oil phase.

The obtained pre-emulsion was ultrasonicated using a UP400S (Ultra-Schallprozessor, Dr. Hielscher GmbH, Teltow, Germany) for 5 min at amplitude of 70%.

### 3.3. SLN Characterization

Mean particles size (Z-Average) and polydispersity index (PDI) of the prepared formulations (SLN and SLN-BXL) were determined by dynamic light scattering using a Zetasizer Nano-ZS90 (Malvern Instrument Ltd., Worcs, UK), equipped with a solid-state laser, with a nominal power of 4.5 mW with a maximum output of 5 mW at 670 nm.

Analyses were performed using a 90° scattering angle at 25 ± 0.2 °C. Zeta potential (ZP) was determined using the electrophoretic light scattering (ELS) technique, which measures the electrophoretic mobility of particles in a dispersed system, indicating its stability. For measurements, each sample were prepared diluting 100 µL of SLN suspension with 900 µL of distilled water. Each value was measured at least in triplicate.

### 3.4. Encapsulation Efficiency

SLN-BXL preparations (0.4 mL) were loaded onto a Sephadex LH20 column (1.0 × 10 cm) eluted in sequence with: water (15 mL), H_2_O:EtOH (50:50; 10 mL), EtOH (10 mL), and acetone (10 mL). In these conditions, the aqueous fraction contains the entrapped BXL, whereas the acetone fraction contains the unentrapped (free) BXL.

The entrapped and free BXL were quantified by HPLC–UV (Agilent, Series 1100) equipped with a diode array detector set at 280 and 390 nm. The chromatographic separation occurred in a Luna C-18 (Phenomenex; 5 μM, 250 mm × 4.60 mm) column employing the conditions previously adopted [19].

The entrapment efficiency % (EE%) was determined according to Equations (1) and (2):

EE% = [(*mgBXL_tot_* − *mgBXL_free_*) ÷ *mgBXL_tot_*] × 100 (1)

EE% = (*mgBXL_entrapped_* ÷ *mgBXL_tot_*) × 100 (2)

### 3.5. BXL Release from SLN

The in vitro release study of BXL was performed employing dialysis tubes with a molecular weight cut-off of 3.5 kDa (Spectra/Pro, Spectrum Lab., Rancho Dominguez, CA, USA) Briefly, 1 mL of SLN-BXL formulations containing 0.6 mg/mL of BXL were placed into a dialysis tube and dispersed in a beaker containing 20 mL of 50 mM citrate buffer (pH 5), 20 mL of 50 mM TRIS buffer (pH 7.4), or 20 mL of H_2_O/EtOH 80:20 mixture.

The solution was stirred at 200 rpm at 37 ± 0.5 °C. At pre-determined intervals within 24 h, 1 mL of the release medium was withdrawn and replaced with an equal volume of the same fresh release medium. The samples were lyophilized and then analyzed by HPLC–UV with the same chromatographic conditions employed for the entrapment efficiency determination.

### 3.6. Cell Culture

HFF-1 were cultured in Dulbecco’s modified Eagle’s medium supplemented with 15% fetal bovine serum, 4.5 g/L glucose, 100 U/mL penicillin, and 100 μg/mL streptomycin. Cells were seeded in 96-well microplates at a constant density (8 × 10^3^ cells/well) to obtain identical experimental conditions in the different tests, and to achieve a high accuracy of the measurements. After 24 h of incubation in a humidified atmosphere of 5% CO_2_ at 37 °C to allow cell attachment, the cells were treated with different concentrations of SLN-BXL (7.3–14.6–29.2 μM) and/or with the corresponding amount of BLX and empty SLN (SLN 1:200; 1:100; 1:50) for 12 h and 24 h. Four replicates were performed for each sample. At the end of the treatment, the cells were scraped, washed with PBS, and immediately utilized for the analysis.

### 3.7. MTT Bioassay

The MTT assay was performed to assess the cells viability. After 24 h of incubation in humidified atmosphere of 5% CO_2_ at 37 °C to allow cell attachment, cells were treated with different concentrations of empty SLN (1:200; 1:100; 1:50), of BLX and SLN-BXL (7.3–14.6–29.2 μM). This assay measures the conversion of tetrazolium salt to yield colored formazan in the presence of metabolic activity. The amount of formazan is proportional to the number of living cells [32].

The optical density was measured with a microplate spectrophotometer reader (Titertek Multiskan, Flow Laboratories, Helsinki, Finland) at λ = 570 nm. Results are expressed as percentage cell viability with respect to control (untreated cells).

### 3.8. ROS Determination

The inhibitory effects of SLN on ROS production were investigated on untreated and treated cells using a fluorescent probe 2′,7′-dichlorofluorescein diacetate (DCFH-DA) [33].

This probe, because of its chemical characteristics, diffuses into the cells: intracellular esterases hydrolyze the acetate groups and the resulting DCFH then reacts with intracellular oxidants resulting in the observed fluorescence. The intensity of fluorescence is proportional to the levels of intracellular oxidant species.

HFF1 cells, pre-treated with different concentrations of SLN (1.200; 1:100; 1:50), of BLX and SLN-BXL (7.3–14.6–29.2 μM) for 180 min, were stimulated with interleukin-2β (IL-2β) (3 μM) for 12 h. After treatments the culture medium was aspirated, the cells washed with PBS, treated with DCFH-DA (5 µM) and DAPI (5 µM) in PSB, and incubated for 15 min. After the time had elapsed, fluorescence was read with a microplate reader (Multiskan EX-Thermolab System) by selecting the following wavelengths: DCF (λ excitation = 493 nm; λ emission = 523 nm) and DAPI (λ excitation = 358 nm; λ emission = 461 nm).

The fluorescence of DCF referred to the amount of ROS normalized for the number of cells by calculating the ratio of DCF fluorescence/DAPI fluorescence, and then the results were expressed as a percentage compared to the untreated control.

### 3.9. Differential Scanning Calorimetry

Calorimetric analysis was performed using a Mettler Toledo STAR^e^ thermoanalytical system (Greifensee, Switzerland) equipped with a DSC822 calorimetric cell. A Mettler TA-STAR^e^ software (version 16.00) (Greifensee, Switzerland) was used to obtain and analyze data. The calorimeter was calibrated using Indium (99.95%), based on the setting of the instrument. The sensitivity was automatically chosen as the maximum possible by the calorimetric system. For this, 160 µL aluminum calorimetric pans were used. Enthalpy changes were calculated from the peak areas.

### 3.10. Empty SLN and SLN-BXL Analysis

To evaluate the thermotropic behavior of the SLN, the formulation was submitted to DSC analysis under N2 flow (70 mL/min) as follows: a heating scan from 5 to 85 °C, at 2 °C/min, and a cooling scan from 85 to 5 °C, at 4 °C/min, at least three times to confirm the reproducibility of data [34].

### 3.11. MLV/SLN Analysis

A total of 30 µL of MLV and 90 µL of SLN or SLN-BXL were placed in a 160 µL DSC aluminum pan, which was hermetically sealed and subjected to calorimetric analysis under N2 flow (70 mL/min) as follows: (1) a heating scan from 5 to 85 °C at the rate of 2 °C/min, (2) a cooling scan from 85 to 37 °C at the rate of 4 °C/min, (3) an isothermal period of one hour at 37 °C, and (4) a cooling scan from 37 to 5 °C (4 °C/min). This procedure was repeated eight times [35].

## 4. Conclusions

The aim of this research was to prepare solid lipid nanoparticles containing a benzo[k,l]xanthene lignan possessing antioxidant properties to be used for topical application. In a precedent study [19], we obtained a SLN preparation with promising results in terms of physicochemical properties, but which did not display an optimal cytotoxicity assessment; for this reason, a new formulation, based on the same solid lipid, was developed.

SLN-BXL show a mean size around 200 nm, PDI of 0.2, and zeta potential of about −28 mV over a period of three months, and an encapsulation efficiency of ≈70%. SLN-BXL are able to penetrate and release the encapsulated compound into a biomembrane model, and show relevant anti-oxidant activity in an in vitro cellular model, and also an optimal double-action release of BXL in the dialysis-bag experiment. Positively, the formulation does not influence the cellular viability, indicating its safety. In conclusion, based on these results, the formulation can be proposed as a candidate for preventive remedies against skin disorders induced by increased levels of ROS.

In future research, given these promising results, we will assess the insertion of the formulation in a secondary pharmaceutical vehicle for topical delivery: we have already conducted some bibliographic scavenging and preliminary experiments that indicate that hydrogel could be a punctual solution, since it has high biocompatibility with human skin, and it has already been used successfully to contain and release SLN suspensions. The hydrogel we will propose is based on a very water-soluble variant of the Carbopol polymer with the addition of MilliQ water, glycerol, and triethanolamine. These components, in adequate ratios and experimental conditions, form a dense, viscoelastic gel with a rich hydrophilic matrix suitable for the entrapment of the SLN suspension. Preliminary tests show how the product has stable rheological parameters, such as viscosity, complex module, and extrapolated yield point, comparable with other well-studied polymeric hydrogel formulations already employed in SLN delivery; these first promising results will be further deepened in a dedicated study.

## Data Availability

Data were generated at the Department of Drug and Health Sciences and at the Department of Chemical Sciences, University of Catania. Data supporting the results of this study are available from the corresponding authors on request.

## Supplementary Materials

The following supporting information can be downloaded at: https://www.mdpi.com/article/10.3390/molecules27185887/s1, Figure S1: Predicted Physicochemical properties and ADME parameters of BXL.
