# Supplementary material for: Benzo[k,l]xanthene Lignan-Loaded Solid Lipid Nanoparticles for Topical Application: A Preliminary Study
Source: Molecules. 2022 Sep 10;27(18):5887. doi: 10.3390/molecules27185887 (PMC9503089; doi:10.3390/molecules27185887)
Supplement: Supplementary file 1 [file molecules-27-05887-s001.zip › molecules-1895402-supplementary.pdf]

Supplementary Material

Benzo[k,l]xanthene Lignan-Loaded Solid Lipid Nanoparticles for Topical Application: A Preliminary Study

Cristina Torrisi [1], Nunzio Cardullo [2], Stefano Russo [1], Alfonsina La Mantia [1], Rosaria Acquaviva [1], Vera Muccilli [2], Francesco Castelli [1] and Maria Grazia Sarpietro [1,*]

[1] Department of Drug and Health Sciences, University of Catania, Viale Andrea Doria 6, 95125 Catania, Italy
[2] Department of Chemical Sciences, University of Catania, Viale Andrea Doria 6, 95125 Catania, Italy
* Correspondence: mg.sarpietro@unict.it

Molecule 1

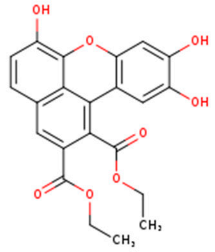

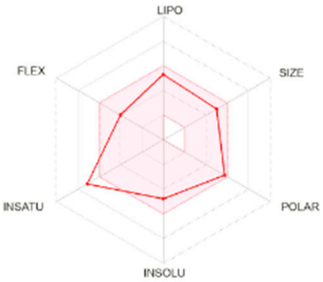

SMILES CCOC(=O)c1c(cc2c3c1cc(O)c(cc1Oc3c(cc2)O)O)C(=O)OCC

Physicochemical Properties	
Formula	C22H18O8
Molecular weight	410.37 g/mol
Num. heavy atoms	30
Num. arom. heavy atoms	16
Fraction Csp3	0.18
Num. rotatable bonds	6
Num. H-bond acceptors	8
Num. H-bond donors	3
Molar Refractivity	107.70
TPSA	122.52 Å²

Lipophilicity	
Log $P_{o/w}$ (iLOGP)	2.72
Log $P_{o/w}$ (XLOGP3)	3.78
Log $P_{o/w}$ (WLOGP)	4.08
Log $P_{o/w}$ (MLOGP)	1.57
Log $P_{o/w}$ (SILICOS-IT)	3.59
Consensus Log $P_{o/w}$	3.15

Water Solubility	
Log *S* (ESOL)	-4.76
Solubility	7.06e-03 mg/ml ; 1.72e-05 mol/l
Class	Moderately soluble
Log *S* (Ali)	-6.05
Solubility	3.69e-04 mg/ml ; 8.98e-07 mol/l
Class	Poorly soluble
Log *S* (SILICOS-IT)	-5.47
Solubility	1.38e-03 mg/ml ; 3.36e-06 mol/l
Class	Moderately soluble

Pharmacokinetics	
GI absorption	High
BBB permeant	No
P-gp substrate	No
CYP1A2 inhibitor	Yes
CYP2C19 inhibitor	No
CYP2C9 inhibitor	Yes
CYP2D6 inhibitor	No
CYP3A4 inhibitor	No
Log K_p (skin permeation)	-6.12 cm/s

Druglikeness	
Lipinski	Yes; 0 violation
Ghose	Yes
Veber	Yes
Egan	Yes
Muegge	Yes
Bioavailability Score	0.55

Medicinal Chemistry	
PAINS	1 alert: catechol_A
Brenk	2 alerts: catechol, more_than_2_esters
Leadlikeness	No; 2 violations: MW>350, XLOGP3>3.5
Synthetic accessibility	3.65

Figure S1. Predicted Physicochemical properties and ADME parameters of BXL [17,18].

17. Daina, A.; Michielin, O.; Zoete, V. SwissADME: A Free Web Tool to Evaluate Pharmacokinetics, Drug-Likeness and Medicinal Chemistry Friendliness of Small Molecules. *Sci. Rep.* **2017**, *7*, 42717, doi:10.1038/srep42717.

18. Daina, A.; Michielin, O.; Zoete, V. ILOGP: A Simple, Robust, and Efficient Description of n-Octanol/Water Partition Coefficient for Drug Design Using the GB/SA Approach. *J. Chem. Inf. Model.* **2014**, *54*, 3284–3301, doi:10.1021/ci500467k.
